# Health Service Experiences of Transgender Individuals from Foreign Backgrounds in Finland

**DOI:** 10.1093/eurpub/ckac129.733

**Published:** 2022-10-25

**Authors:** M Czimbalmos, S Rask

**Affiliations:** Equality-unit, The Finnish Institute for Health and Welfare, Helsinki, Finland

## Abstract

**Background:**

Internationally, an increasing body of scholarship has focused on the experiences of transgender individuals when accessing gender-affirming healthcare. However, the experiences of transgender individuals who belong to the foreign background population in Finland have rarely been studied. This study aims to fill the gap in research and contribute to the understanding of the experiences of acquiring gender-affirming healthcare among those, who fall into the intersections of transness and also identify of foreign origin in Finland.

**Methods:**

Fourteen semi-structured qualitative interviews were conducted and analyzed with reflexive thematic analysis (RTA), through the framework of intersectionality. The interviews were part of a broader sample of qualitative data, collected about the experiences of sexual and gender minorities among the foreign origin populations in Finland.

**Results:**

The analysis showed two main interconnected themes. Firstly, perceived barriers when accessing gender-affirming care. In this theme, the intersections of transgender identity, foreign background, class, and age affected the experiences of the individuals. Secondly, the necessity of “performing identities:” the intersections of class, transgender identity, and race affected those.

**Conclusions:**

The findings of the current study suggest that the intersectional aspects of individual identities create structural inequalities in the Finnish gender-affirmation healthcare system. To tackle these inequalities, further research is needed on the healthcare experiences of gender minorities in Finland both within and outside the scope of transgender-specific healthcare.

**Key messages:**

• Intersectional aspects of individual identities create structural inequalities in accessing gender-affirming healthcare.

• Further research is needed on the healthcare experiences of gender minorities that examines health and wellbeing using an intersectional lens.

